# Impact of the sequential implementation of a pharmacy-driven methicillin-resistant *Staphylococcus aureus* (MRSA) nasal-swab ordering policy and vancomycin 72-hour restriction protocol on standardized antibiotic administration ratio (SAAR) data for antibiotics used for resistant gram-positive infections

**DOI:** 10.1017/ice.2023.190

**Published:** 2024-02

**Authors:** Natasha N. Pettit, Cynthia T. Nguyen, Alison K. Lew, Jennifer Pisano

**Affiliations:** 1 Department of Pharmacy, University of Chicago Medicine, Chicago, IL; 2 Department of Medicine, University of Chicago Medicine, Chicago, IL

## Abstract

**Objective:**

Vancomycin is often initiated in hospitalized patients; however, it may be unnecessary or continued for longer durations than needed. Oversight of all vancomycin orders may not be feasible with widespread prescribing and strategies to enlist other clinicians to serve as stewards of vancomycin use are needed. We implemented 2 sequential interventions: a protocol in which the pharmacist orders MRSA nasal swab followed by a protocol requiring approval from pharmacists to continue vancomycin for >72 hours.

**Methods:**

In this single-center, retrospective, quasi-experimental study, we evaluated vancomycin use after implementation of a pharmacy-driven MRSA nasal-swab ordering protocol and a vancomycin 72-hour restriction protocol. The primary outcome was the change in the standardized antibiotic administration ratio (SAAR) for antibacterial agents for resistant gram-positive infections. We also evaluated the impact on antibiotic utilization.

**Results:**

Following the MRSA swab protocol, the SAAR decreased from 1.26 to 1.13 (*P* < .001; 95% confidence interval [CI], 1.16–1.25). After the 72-hour approval process, the SAAR was 0.96 (*P* < .001; 95% CI, 1.0–1.12). Vancomycin utilization decreased from 138.9 to 125.3 days of therapy per 1,000 patient days following the MRSA swab protocol (*P* < .001) and to 112.7 (*P* < .001) following the 72-hour approval protocol. Interrupted time-series analysis identified a similar rate of decline in utilization following the 2 interventions (−0.3 and −0.5; *P* = .16). Both interventions combined resulted in a significant reduction (−1.5; *P* < .001).

**Conclusion:**

Implementation of a pharmacist-driven MRSA nasal-swab ordering protocol, followed by a 72-hour approval protocol, was associated with a significant reduction in the SAAR for antibiotics used in the treatment of resistant gram-positive infections and a reduction in vancomycin utilization. Leveraging the oversight of primary service clinical pharmacists through these protocols proved to be an effective strategy.

Vancomycin is commonly prescribed empirically for hospitalized patients to provide broad-spectrum gram-positive coverage, including methicillin-resistant *Staphylococcus* aureus (MRSA). However, use is inappropriate >25% of the time, with the most common reasons being excessive durations or lack of documented infection.^1^ Inappropriate prescribing of vancomycin unnecessarily exposes patients to severe antibiotic toxicities (eg, nephrotoxicity, thrombocytopenia) and strains hospital resources (eg, nursing time, pharmacy resources, laboratory resources).

Stewardship initiatives targeting the inappropriate prescribing of vancomycin, including pre-authorization, prospective audit with feedback, MRSA screening, and the completion of antibiotic timeouts, are potential strategies to optimize use.^2,3^ Implementation of such strategies may be less feasible at large medical centers or programs with limited resources, where the volume of vancomycin orders may exceed the capabilities of stewardship programs. To accomplish effective stewardship of vancomycin prescribing, it may be necessary to leverage the oversight of clinicians outside the stewardship program to act as stewardship extenders. Clinicians with more direct interactions with the medical teams or providers prescribing antibiotics, such as clinical pharmacists, may be more effective at encouraging discontinuation of unnecessary antibiotics as well.^4^

After reviewing a random sampling of vancomycin regimens at our medical center, we observed that although empiric initiation of vancomycin was generally appropriate, the mean duration of empiric vancomycin was 4–5 days. We also observed that our standardized antimicrobial administration ratio (SAAR) for antibacterial agents against resistant gram-positive infections was >1, signaling that our use of agents targeting resistant gram-positive organisms is higher than that of our peers. We then compared our vancomyin utilization with other centers for benchmarking analysis, and vancomycin use was higher than that of other centers. These findings formed the basis for why we chose to implement interventions targeting reduction of vancomycin utilization. On May 1, 2019, we created a protocol allowing pharmacists to order MRSA nasal swabs for patients started on anti-MRSA therapy. On February 1, 2020, we implemented a protocol requiring providers to obtain approval from primary team clinical pharmacists to continue empiric vancomycin beyond 72 hours, with stewardship pharmacists available for case review as needed.

## Methods

In this retrospective, single-center (811-bed medical center in an urban setting), quasi-experimental study, we evaluated the impact of a protocol to reduce empiric vancomycin utilization by implementing a pharmacist-to-order MRSA nasal-swab protocol followed by a 72-hour vancomycin approval process targeting regimens continued beyond 72 hours. Only adult inpatients 18 years or older were included. We included 3 periods in our analysis: (1) pre-MRSA swab protocol from May 1, 2018, to April 30, 2019, (2) post-MRSA swab protocol from May 1, 2019, to January 31, 2020, and (3) post-MRSA swab protocol plus the 72-hour protocol from February 1, 2020, to January 31, 2022. The primary end point was the standardized antimicrobial administration ratio (SAAR) for antibacterial agents targeting resistant gram-positive infections. This SAAR category includes vancomycin, linezolid, daptomycin, ceftaroline, dalbavancin, oritavancin, tedizolid, quinupristin-dalfopristin, and telavancin.^5^ Among these agents, only vancomycin, linezolid, daptomycin, ceftaroline, and quinupristin-dalfopristin are on inpatient formulary at our medical center. For the secondary outcome, we determined overall utilization defined as days of therapy per 1,000 patient days (DOT/1,000 PD) for these formulary agents to identify the primary driver for the change in the SAAR.

### Intervention

On May 1, 2019, a protocol was approved that allowed all pharmacists to place orders for MRSA nasal swabs for any patient started on anti-MRSA therapy. Although the initial policy allowed pharmacists to order the MRSA nasal swab only when the indication for antibiotics was for pneumonia, this was later expanded to all indications in October 2021. Our medical center performs MRSA nasal swabs using culture-based methodology with a turnaround time of 2–3 days. Beginning February 1, 2020, to continue vancomycin beyond 72 hours, providers were required to obtain approval from the clinical pharmacist assigned to the service. There was no automatic stop on the order; rather, the pharmacist would discuss with the provider at 72 hours if therapy was appropriate to continue. If vancomycin was continued, a note was placed by the pharmacist in the chart outlining the indication for continuation of therapy. Complicated cases or cases in which there was disagreement between the pharmacist and provider regarding the need for vancomycin continuation were reviewed by an antimicrobial stewardship pharmacist for further discussion. This process was carried out on weekdays and weekends, with the caveat that on weekends or holidays, if unable to communicate with the providers (eg, pages not answered or provider was unavailable for discussion) then the review could be delayed until the next business day. Prior to and throughout the implementation of these protocols, pharmacists assigned to clinical services were responsible for reviewing all vancomycin orders under a pharmacokinetics consultation service protocol and performed 48- to 72-hour antibiotic timeouts for all antibiotics.

Our inpatient adult antimicrobial stewardship (ASP) team is comprised of 3 pharmacists and 1 infectious diseases physician. Of the 3 ASP pharmacists, 1 pharmacist covers the day-to-day stewardship activities at any one time, including pre-authorization of restricted agents, postprescriptive audit and feedback, and culture review. Clinical pharmacists who cover the various medical service lines at our hospital are responsible for vancomycin dosing and monitoring through our pharmacokinetics consultation service protocol. Our clinical pharmacists are active members of the interdisciplinary rounding teams and contribute to daily discussions regarding medication therapy, including antibiotic regimens.

### Statistical analysis

The NHSN statistics calculator was utilized to assess the difference in SAAR values. The NHSN uses a mid-*P* exact test (based on Poisson distribution) providing a 2-tailed *P* value and 95% confidence interval (CI). An interrupted time-series analysis was also completed using Stata version 14.0 statistics and data analysis software (StataCorp, College Station, TX) to estimate the effect of the 2 components of the intervention on vancomycin utilization. A 2-sample *t* test was performed to assess differences in antimicrobial utilization.

## Results

**Figure 1.**
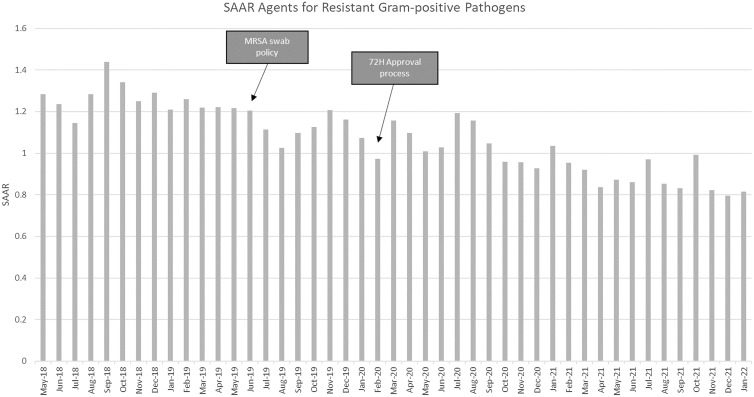
Monthly standardized antimicrobial administration ratio (SAAR) for antibiotics active against resistant gram-positive organisms.

The SAAR for antibacterial agents for resistant gram-positive infections for the year prior to any intervention was 1.26. Following the implementation of the MRSA swab protocol at 9 months (post-MRSA swab), the SAAR for this category of antibiotics was 1.13 (*P* < .001; 95% CI, 1.16–1.25). At 2 years following the addition of the 72-hour approval process, the SAAR was 0.96 (*P* < .001; 95% CI, 1.0–1.2). The monthly SAAR data for agents used to treat resistant gram-positive infections are shown in Figure 1.

**Figure 2.**
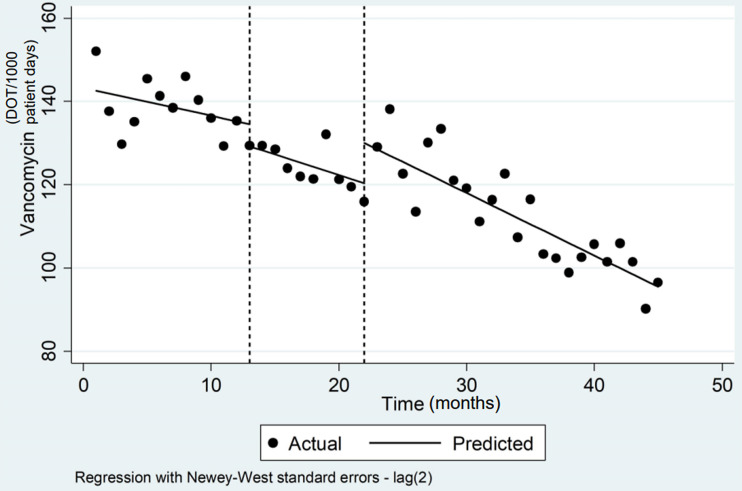
Interrupted time-series analysis estimating the impact on vancomycin utilization of each component of the intervention (MRSA swab policy followed by 72-hour approval process). The vertical dashed-lines at time point ‘13’ and ‘22’ represent the 2 interventions. Time point 13 represents when the MRSA swab policy was implemented, and time point 22 indicates when the 72-hour approval process was added.

For the secondary outcome of antimicrobial utilization, the DOT/1,000 PD for vancomycin was reduced from 138.9 (1-year prior to any intervention) to 125.3 (*P* < .001) 9 months following the MRSA nasal-swab protocol. At 2-years following the implementation of the 72-hour approval process, utilization was further reduced to 112.7 (*P* < .001). The *P* values provided are relative to initial utilization prior to any intervention. On interrupted time-series analysis, the rate of change following the second intervention was similar to the rate of change in utilization following the first intervention (−0.3 to −0.5; 95% CI, −1.3 to 0.22; *P* = .16) (Table 1 and Figure 2). The change in utilization over time between the MRSA swab policy and 72-hour approval was statistically significant (−0.98; 95% CI, −1.5 to −.46; *P* = .001), as was the change over time following the addition of the 72-hour approval process (−1.5; 95% CI, −1.9 to −1.1; *P* < .001), when compared to the pre-intervention period. The utilization of other broad-spectrum agents with activity against resistant gram-positive infections did not change significantly following the MRSA swab protocol or 72-hour approval process, suggesting that the reduction in vancomycin utilization was the primary factor in reducing the SAAR for antimicrobials used for resistant gram-positive infections. Combined, utilization of these other agents was 7.95 at 1 year prior to any intervention, 7.81 at 9 months after the MRSA swab protocol (*P =* .87), and 7.52 at 2 years following the 72-hour approval process (*P =* .53).

**Table 1.** Interrupted Time-Series Analysis Results

Variable	Coefficient	95% CI	*P* Value
Level change after intervention 1^a^	−5.4	−12 to 1.3	.10
Trend/slope change after intervention 1	−0.32	−1.5 to 0.86	.60
Trend/slope change between interventions 1 and 2^b^	−0.99	−1.5 to −0.46	.001
Level change after intervention 2	9.7	3.9 to 15.4	.002
Trend/slope change after intervention 2	−0.52	−1.3 to 0.22	.16
Trend/slope change following intervention 2	−1.5	−1.9 to −1.1	<.001

Note. CI, confidence interval.

a Intervention 1: MRSA swab policy.

b Intervention 2: 72-hour vancomycin approval.

## Discussion

The sequential implementation of a protocol enabling pharmacists-to-order MRSA nasal swabs followed by a protocol requiring clinical pharmacist approval to continue empiric vancomycin beyond 72 hours was an effective strategy to reduce overall vancomycin utilization at our medical center. We observed a sustained reduction in use at 2 years following protocol implementation. Both the SAAR for antibiotics used for resistant gram-positive infections and overall utilization in DOT/1,000 PD was significantly reduced following the interventions.

We introduced a protocol allowing pharmacists to place orders for MRSA nasal swabs for patients started on anti-MRSA therapy 9 months prior to the implementation of the 72-hour approval process, which resulted in an initial reduction in overall utilization. However, this intervention alone did not result in a significant reduction in our SAAR. Prior studies have shown a significant reduction in vancomycin durations of therapy and utilization with pharmacist-driven MRSA nasal-swab protocols, given the high negative predictive value.^3^

Following the implementation of the protocol allowing pharmacists to order MRSA nasal swabs to assess for MRSA colonization, we added a 72-hour approval protocol requiring pharmacists and prescribers to perform an antibiotic timeout to assess continued need for vancomycin beyond 72 hours. Antibiotic timeouts performed after 48–72 hours of initiating empiric antibiotic regimens may be effective in optimizing antimicrobial therapy; they are a strategy recommended by the CDC Core Elements for Antimicrobial Stewardship.^4,6,7^ In previous studies, antibiotic timeouts in the hospital setting involving vancomycin have been shown to be associated with cost-savings, increased rates of discontinuation, shorter durations of therapy, and reductions in utilization.^2,7–10^ Manigaba et al^10^ implemented a strategy in which a 72-hour timeout for vancomycin orders was completed by pharmacists who recommend discontinuation or consultation with the infectious diseases team. To our knowledge, this is the first report of implementing such a protocol to reduce vancomycin utilization and to provide data using the SAAR and DOT/1,000 PD as metrics.

Protocol implementation and our ability to assess the impact on antimicrobial use at our medical center demonstrated the value of submitting data on antimicrobial use to the National Healthcare Safety Network (NHSN) antimicrobial use (AU) module. Submitting data to the NSHN enables medical centers to assess their utilization trends and benchmark use against similar medical centers with SAAR values for certain types or groups of antimicrobials. The SAAR is a ratio of observed antibiotic use relative to predicted use based on models using certain facility characteristics such as location type, facility type, teaching status, number of hospital beds, number of ICU beds, and average hospital length of stay.^5^ An SAAR <1 indicates that use is less than predicted or less than peer hospital use, and an SAAR >1 indicates that use is above predicted or greater than peer hospital use. Although a SAAR above or below 1 indicates a difference in utilization compared to peer medical centers, it does not necessarily mean that observed use is inappropriate. Additional investigation is needed to assess whether the variance in use compared to peer hospitals requires an intervention. Notably, when assessing SAAR data that have been risk adjusted to a benchmark population to evaluate the impact of an intervention, trend analyses (eg, interrupted time-series analysis) are not recommended. Guidance from the NHSN on using SAAR data recommends against trend analyses to determine whether there has been a statistically significant change in utilization across many points in time because the proportionality assumption is unlikely to hold true across many time points.^5^

When we observed an SAAR >1 for the included category of antibiotics, we conducted a targeted review of vancomycin orders and identified that durations of empiric vancomycin needed optimization. This was the impetus for enacting a pharmacist-to-order MRSA screen protocol and for creating a protocol requiring clinical pharmacist approval to continue empiric vancomycin orders beyond 72 hours. As a result of these protocols, the SAAR value for antibiotics used for resistant gram-positive infections was significantly reduced to a value <1, suggesting that our utilization is now in line with or slightly below that of our peers. The NHSN data facilitate the identification of high level and evaluable stewardship interventions. Other examples of how stewardship programs can utilize the NHSN AU module to identify and measure targeted interventions have been reported as well.^11^

This study had several limitations. The study period included the COVID-19 pandemic. It is well known that many hospitalized patients presenting with COVID-19 received empiric antibiotics, primarily with third-generation cephalosporins, macrolides, and fluoroquinolones.^12,13^ We were not able to provide details on how often MRSA nasal swabs were ordered by pharmacists among those receiving at least 72 hours of vancomycin. We were not able to determine the numbers of patients for whom vancomycin was approved versus not approved by pharmacists following the 72-hour review nor how often antimicrobial stewardship pharmacists had to be consulted to review the case. Anecdotally, the antimicrobial stewardship pharmacist was infrequently consulted. Notably, the 72-hour vancomycin approval process is a required component of our clinical pharmacist daily activities, so it is expected that all patients that have received >72 hours of vancomycin therapy have undergone this review. We also assumed in our SAAR analysis that vancomycin was the primary driver in the SAAR value for antimicrobials with activity against resistant gram-positive organisms. This assumption was accurate in that utilization of the other antibiotics used in this SAAR category ranged from 7.5 to 7.9 DOT/1,000 PD, compared to vancomycin, which ranged from 112 to 138 DOT/1,000 PD. We did not assess clinical outcomes in this study to determine whether this strategy posed a detriment to patient outcomes or safety. However, previous studies evaluating the impact of MRSA screening protocols and antibiotic timeout processes that had facilitated higher rates of discontinuation of vancomycin and other antibiotics reported no negative effects on outcomes such as mortality and readmissions.^2,14^ Last, we implemented the 72-hour approval protocol 9 months following the MRSA nasal-swab protocol, giving us a shorter window of time to evaluate the impact on the SAAR and antimicrobial utilization of this one protocol alone. Finally, this timeline may not have been long enough to assess the true impact of the MRSA nasal swab protocol alone.

The sequential implementation of a pharmacist-to-order MRSA nasal swabs protocol and an antimicrobial stewardship supported protocol requiring approval to continue empiric vancomycin beyond 72 hours empowered our clinical pharmacy team to effectively regulate vancomycin durations of therapy. Involving frontline clinicians, such as clinical pharmacists, in stewardship efforts to optimize antimicrobial utilization is an effective strategy.
